# Multidimensional Scaling Reveals the Main Evolutionary Pathways of Class A G-Protein-Coupled Receptors

**DOI:** 10.1371/journal.pone.0019094

**Published:** 2011-04-22

**Authors:** Julien Pelé, Hervé Abdi, Matthieu Moreau, David Thybert, Marie Chabbert

**Affiliations:** 1 CNRS UMR 6214 – INSERM 771, Faculté de Médecine, Angers, France; 2 School of Behavioral and Brain Sciences, The University of Texas at Dallas, Richardson, Texas, United States of America; University of South Florida College of Medicine, United States of America

## Abstract

Class A G-protein-coupled receptors (GPCRs) constitute the largest family of transmembrane receptors in the human genome. Understanding the mechanisms which drove the evolution of such a large family would help understand the specificity of each GPCR sub-family with applications to drug design. To gain evolutionary information on class A GPCRs, we explored their sequence space by metric multidimensional scaling analysis (MDS). Three-dimensional mapping of human sequences shows a non-uniform distribution of GPCRs, organized in clusters that lay along four privileged directions. To interpret these directions, we projected supplementary sequences from different species onto the human space used as a reference. With this technique, we can easily monitor the evolutionary drift of several GPCR sub-families from cnidarians to humans. Results support a model of radiative evolution of class A GPCRs from a central node formed by peptide receptors. The privileged directions obtained from the MDS analysis are interpretable in terms of three main evolutionary pathways related to specific sequence determinants. The first pathway was initiated by a deletion in transmembrane helix 2 (TM2) and led to three sub-families by divergent evolution. The second pathway corresponds to the differentiation of the amine receptors. The third pathway corresponds to parallel evolution of several sub-families in relation with a covarion process involving proline residues in TM2 and TM5. As exemplified with GPCRs, the MDS projection technique is an important tool to compare orthologous sequence sets and to help decipher the mutational events that drove the evolution of protein families.

## Introduction

Proteins with a seven transmembrane helix scaffold are widespread in the animal kingdom and are usually assumed to be G-protein-coupled receptors (GPCRs) by similarity with their vertebrate counterparts. Because they transduce signals from a wide variety of chemical or physical stimuli, these receptors are involved in the perception by the cell of its environment and the regulation of most physiological functions [1]. Impaired GPCR signaling characterizes numerous pathologies of the cardiovascular, immune, neurological and metabolic systems. Consequently, GPCRs constitute major therapeutic targets for a wide spectrum of diseases and are subject to intensive investigation aimed at drug discovery.

GPCRs are classified into several classes whose common origin is still debated [2], [3]. Within each class, however, receptors are clearly phylogenetically related and share conserved sequence patterns. With about 300 non-olfactory and 400 olfactory members, class A or rhodopsin-like GPCRs represent up to 90% of human GPCRs. Non-olfactory receptors can be further classified into a dozen of sub-families. However, the hierarchy of these sub-families is still unresolved and there is a strong discrepancy between the conclusions of different studies [2], [4], [5], [6]. Understanding the mechanisms that led to the diversification of this family would help decipher the specificity of the sequence-structure-function relationships of each sub-family and would improve drug design targeted to GPCRs.

The phylogeny of a huge family of proteins such as GPCRs is far from obvious. Most current phylogenetic methods implicitly assume that the sequences can be classified according to a binary tree and try to reconstruct this tree. However, evolution may proceed either by bifurcation or by radiation. Radiative evolution, which should be described by polytomic trees, may account for discrepancies between binary trees [7], [8]. In addition, evolution works on the sequence level, but proceeds under strong structural and functional constraints. As a consequence, selective pressure on a given amino acid may depend on the identity of amino acids at other sites, resulting in correlated mutations and/or branch specific changes in evolutionary rates [9], [10], [11]. This so-called covarion process may lead to misinterpretation of parallel/convergent evolution and is responsible of topological biases [12], [13]. These difficulties inherent to phylogenetic methods prompted us to consider alternative methods to gain information on the relationships between GPCRs.

One such method is metric multidimensional scaling analysis (MDS) [14], [15], [16]. MDS, also called Principal COordinates analysis (PCO), is an exploratory multivariate procedure designed to identify patterns in a distance matrix. In this regard, when applied to sequences, MDS can be compared to neighbor-joining or UPGMA methods. However, in these methods, sequences are considered by pairwise progression to establish a binary tree, whereas, in MDS, sequences are considered all at once, to determine a sequence space. In that case, sequences are represented, in a low-dimensional Euclidean space, by points whose respective distances best approximate the original distances. In addition, the MDS technique allows the projection of supplementary elements onto a reference or “active” space which is the space defined by the set of the data under scrutiny [15], [17], [18]. The projection technique allows a straightforward comparison of the active and supplementary data and therefore can be used to compare orthologous sequence sets.

In this article, we use MDS to explore the sequence space of class A GPCRs. To interpret patterns in relation with evolution, we projected GPCR sequences from distant species onto the active space of human GPCRs. Applied for the first time to protein sequences, this projection technique helps decipher the factors underlying the evolution of GPCRs.

## Results

### 1. The sequence space of human GPCRs

**Figure 1:**
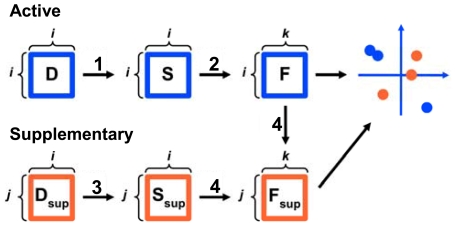
Schematic representation of the MDS analysis. The analysis of *N* active and *N*_sup_ supplementary sequences are represented in blue and orange, respectively. **D** and **D_sup_** represent distance matrices, **S** and **S_sup_** cross-product matrices and **F** and **F_sup_** factor score matrices. The coordinate of the *i* ^th^ active sequence on the *k* ^th^ principal component is directly obtained from the *i* ^th^ element of the *k* ^th^ column of **F**. The coordinate of the *j* ^th^ supplementary sequence on the *k* ^th^ principal component of the active space is directly obtained from the *j* ^th^ element of the *k* ^th^ column of **F_sup_**. The numbers above the arrows refer to the equations given in the Methods section.

In H. sapiens, non-olfactory class A G-protein-coupled receptors (thereafter GPCRs) form a non-redundant set of 283 sequences that are referred to as the active sequence set. Most of these sequences (93%) can be classified into the twelve sub-families listed in Table 1. From the multiple sequence alignment (MSA) of the active sequences, we computed a matrix of pairwise distances, based on sequence identity. Then, the distance matrix was analyzed by MDS, according to the procedure detailed in the Methods section. Briefly, MDS transforms the distance matrix **D** into a cross-product matrix **S** whose eigendecomposition is used to compute a factor score matrix **F** (Figure 1). This last matrix, in turn, gives the coordinates of the active sequences in the active space formed by the eigenvectors (also called principal components) of **S**.

**Table 1:** Summary of the human GPCR set.

Group	Sub-family	Description	Pro in TM2	TM2 Pro position	Pro in TM5	WXFG motif
G0	PEP	Peptide receptors	+++	**2.59**	+++	++
	OPN	Opsins	++	2.59	+++	+++
	MTN	Melatonin receptors	+++	**2.59**	+++	++
G1	SO	Somatostatin/opioid receptors	+++	**2.58**	+++	+++
	CHEM	Chemotactic receptors	+++	**2.58**	+++	+++
	PUR	Purinergic receptors	+++	**2.58**	+++	++
G2	AMIN	Amine receptors	+++	**2.59**	+++	++
	AD	Adenosine receptors	+++	**2.59**	+++	–
G3	LGR	Leucine-rich repeat receptors	–	–	–	Δ
	MEC	Melanocortin, S1P and cannabinoid receptors	–	–	–	–
	PTG	Prostaglandin receptors	++	2.59	–	+
	MRG	MAS-related receptors	–	–	+	–

Human non-olfactory class A GPCRs were assigned to twelve sub-families according to the detailed classification reported in [6], except for the split of the MECA receptors into the AD and MEC sub-families. 7% of the human receptors could not be classified. The symbols indicate the percent of sequences with the pattern considered in human GPCRs (–, +, ++ and +++ correspond to 0%, 0 to 50%, 50 to 80% and ≥80%, respectively). Proline was searched for from position 2.58 to 2.60 in TM2 and at position 5.50 in TM5. The main proline position in TM2 is italic, normal and bold when it is observed in < 50%, 50 to 80% and ≥ 80% of the sequences. Δ indicates that the WXFG motif is shifted to positions 3.19–3.22.

**Figure 2:**
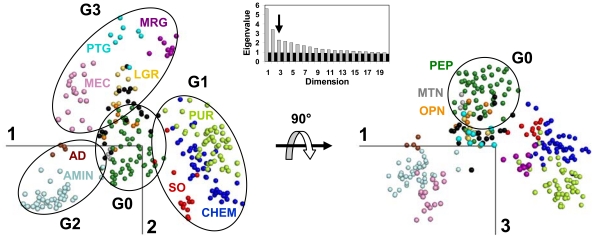
MDS representation of human non-olfactory class A GPCRs. Data are projected onto the planes defined by the first and the second components (left) and by the first and the third components (right). The insert displays the scree plot of the first twenty eigenvalues obtained from the MDS analysis of human GPCRs (grey bars) and, for comparison, the eigenvalues obtained from a random MSA with the same characteristics as human GPCRs (black bars). The color code refers to the GPCR sub-families (AD: brown; AMIN: light blue; CHEM: dark blue; LGR: yellow; MEC: pink; MTN: grey; MRG: violet; OPN: orange; PEP: dark green; PTG: cyan; PUR: light green; SO: red; UC: black).

We can map the sequence space of the human GPCRs onto the 3D space formed by the three components with the largest eigenvalues. For clarity purpose, Figure 2 shows their projection onto the planes formed by the first and second components and by the first and third components. The MDS representation reveals a non-uniform distribution of human GPCRs. The receptors have a radial organization and cluster along a few privileged directions. This organization yields a straightforward classification of the receptors into four groups (named G0 to G3), at an intermediate level between the class and the sub-family levels (Table 1).

**Figure 3:**
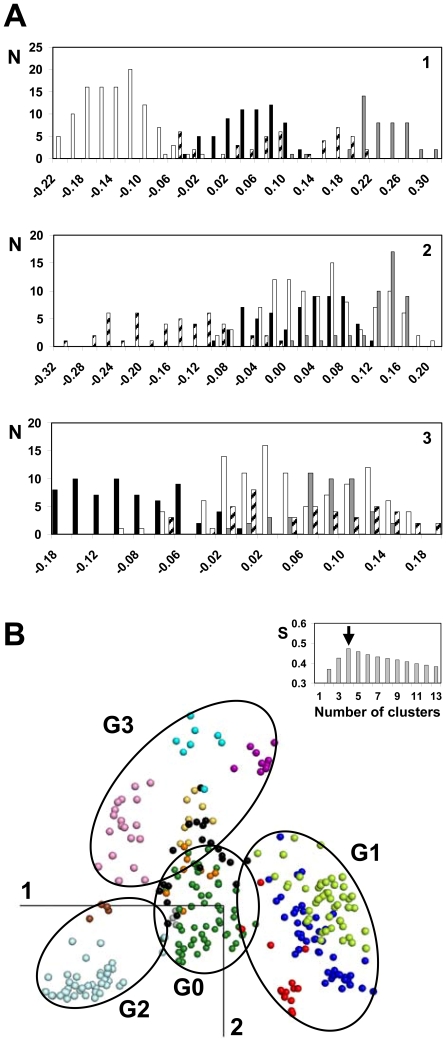
Clustering of human GPCRs. In (A), the histograms show the distribution of the receptors from groups G0 (black bars), G1 (white bars), G2 (grey bars) and G3 (hatched bars) as a function of their coordinates on the first, second and third components (from top to bottom). In (B), the four clusters obtained by *K*-means analysis are visualized by spanning ellipses onto the plane formed by the first two components. The insert displays the Silhouette score *S* obtained for *K*-means clustering as a function of the number of clusters. The color code refers to the GPCR sub-families (AD: brown; AMIN: light blue; CHEM: dark blue; LGR: yellow; MEC: pink; MTN: grey; MRG: violet; OPN: orange; PEP: dark green; PTG: cyan; PUR: light green; SO: red; UC: black).

The first dimension differentiates groups G1 and G2 from the remaining receptors (Figure 3A). Group G1 is characterized by negative coordinates on the first component. It is composed of the SO, CHEM, and PUR sub-families which are phylogenetically related [6]. These three sub-families are separated in the 3D space by a combination of the three components (Figure 2). Group G2 is characterized by positive coordinates on the first component. It includes the AMIN and AD receptors. The second dimension differentiates group G3 whose members have negative coordinates on this axis (Figure 3A). Group G3 includes the LGR, PTG, MRG and MEC sub-families. Finally, the receptors that are most central in the plane formed by the first two components are differentiated by the third component (Figure 3A). This group, named G0 for its central position, includes the PEP, MTN, and OPN sub-families, with these latter two sub-families located on the edges of the group. Unclassified receptors (7% of the human set) cluster either with G0 or with G3.

This intuitive clustering based on visual inspection is corroborated by *K*-means analysis (Figure 3B). The maximum of the Silhouette score [19] is reached for four clusters (Figure 3B, insert), which correspond to the best description of the data. Receptors are attributed to the same clusters by *K*-means and visual inspection, except a few receptors (about 4%) located at the interface between two groups. For the forthcoming analysis, these receptors are assigned to the group including most members of their sub-family.

The only exception for the assignment of a sub-family to a single cluster is observed for the MECA (melanocortin, S1P, cannabinoid and adenosine) receptors. We and others considered these receptors as forming a single sub-family from phylogenetic data [2], [6], but the MDS analysis clearly divides the MECA receptors into two subsets. The adenosine receptors (AD) cluster with the AMIN receptors, as observed in some phylogenetic studies [4], [5], whereas the remaining receptors (MEC), whose coordinates on the second component are negative, cluster with group G3.

The scree plot of the eigenvalues (Figure 2, insert) shows a sharp drop from the first to the third component, followed by a slow decrease towards values similar to those obtained from the MDS analysis of a random multiple sequence alignment with the same characteristics as human GPCRs. This indicates that the first two or three components are sufficient to adequately describe the data and that lower ranking components are not interpretable [20]. Interestingly, groups G0 and G3 form a continuum, but do not overlap significantly on the second dimension (Figure 3B). Most details are thus adequately described by the first two components in agreement with the scree plot. However, the third component improves the discrimination performance, clearly separates groups G0 and G3, and provides a more detailed view of the GPCR space.

### 2. Evolutionary drift of GPCRs

To understand the organization of the sequence space of human GPCRs, we projected additional sets of sequences (referred to as supplementary sequences) onto the space of the active sequences analyzed by MDS (Figure 1). As we are interested in the evolution of sub-families present in humans, supplementary sequences correspond to GPCRs from these sub-families in four selected species. These species have fully sequenced genomes and belong to the cnidarian (*N. vectensis*), nematode (*C. elegans*), chordate (*C. intestinalis*) and verbebrate (*D. rerio*) lineages. Five sub-families (PEP, AMIN, LGR, OPN and SO) are present from cnidarians to vertebrates whereas the other sub-families appeared in bilaterians (AD), chordates (MEC, PTG, CHEM, MTN), vertebrates (PUR) and mammalian (MRG) [6], [21]. Supplementary sequences were aligned against the MSA of human GPCRs and the matrix of distances between supplementary and active sequences was calculated from sequence identity. This supplementary distance matrix was transformed as described in the Methods section to obtain the coordinates of the supplementary sequences when they are projected onto the human active space.

**Figure 4:**
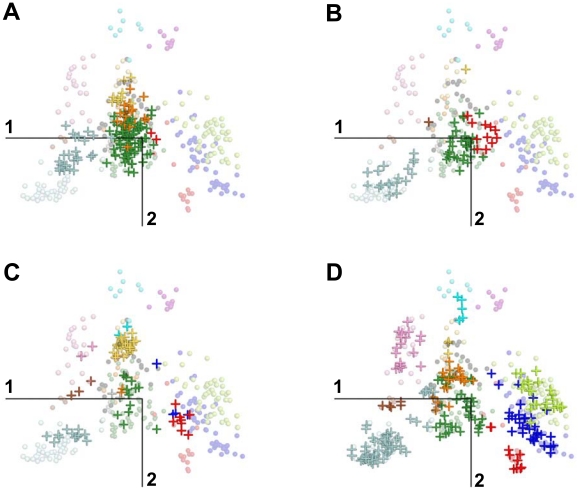
Projection of supplementary GPCR sequences onto the sequence space of human GPCRs. GPCRs from *N. vectensis* (A), *C. elegans* (B), *C. intestinalis* (C) and *D. rerio* (D) are projected onto the plane formed by the first two components of the human active space. Transparent circles and crosses represent human and supplementary elements, respectively. The color code refers to the GPCR sub-families (AD: brown; AMIN: light blue; CHEM: dark blue; LGR: yellow; MEC: pink; MTN: grey; MRG: violet; OPN: orange; PEP: dark green; PTG: cyan; PUR: light green; SO: red; UC: black).

**Figure 5:**
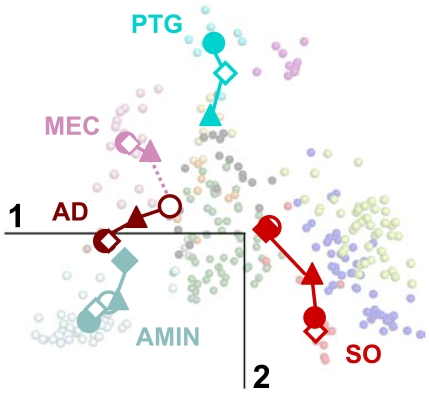
Evolutionary drift of specific sub-families. The barycenters of the SO (red), AMIN (light blue), AD (brown), MEC (pink) and PTG (cyan) sub-families are projected onto the plane formed by the first two components of the human active space. The symbol code indicates the species (*N. vectensis*: closed diamonds, *C. elegans*: open circles; *C. intestinalis*: closed triangles; *D. rerio*: open diamonds*; H. sapiens*: closed circles). The color lines joining the barycenters are given for clarity purpose. The pink dashed line indicates the putative phylogenetic relationship between AD and MEC receptors. Transparent circles represent human elements, with color code referring to the GPCR sub-families (AD: brown; AMIN: light blue; CHEM: dark blue; LGR: yellow; MEC: pink; MTN: grey; MRG: violet; OPN: orange; PEP: dark green; PTG: cyan; PUR: light green; SO: red; UC: black).

The projection of supplementary GPCRs allows the straightforward monitoring of the evolutionary drift undergone by some sub-families while other sub-families remained stable (Figure 4–5). The central position of the PEP receptors is maintained throughout species while no significant shift is observed for the OPN, LGR and MTN receptors. On the other hand, the drift of the AMIN receptors is obvious when comparing the position of this sub-family in *N. vectensis* and vertebrates. The drift of the SO receptors is still more striking because they move from the right side of G0 in *N. vectensis* and *C. elegans* to an intermediate position in *C. intestinalis* and to their final position in vertebrates (Figure 4–5).

The first members of the CHEM sub-family appeared with chordates. In *C. intestinalis*, the members of the CHEM sub-family are not clearly separated from the SO receptors, either by MDS analysis (Figure 4) or by phylogenetic analysis [6]. In vertebrates, the ancestral SO group diverged into three sub-families: “modern” SO, CHEM and PUR receptors. The position of these later ones suggests that they evolved from ancestors of CHEM receptors.

The AD receptors are close to G0 in *C. elegans* and move towards the AMIN receptors in vertebrates. Interestingly, compared to the position of the single AD receptor from *C. elegans*, the AD and MEC receptors from *C. intestinalis* are translated along the first and second components, respectively. Finally, the PTG receptors shift along the second component from chordates to mammalians (Figure 5).

**Figure 6:**
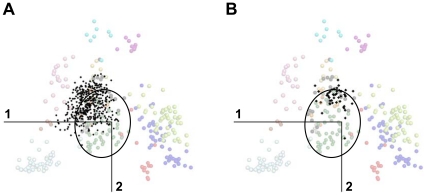
Projection of GPCRs from non-human sub-families onto the sequence space of human GPCRs. GPCRs from *N. vectensis* (A) or *C. elegans* (B) that cannot be attributed to sub-families present in humans are projected onto the plane formed by the first two components of the human active space. Projected elements (397 and 47 sequences from *N. vectensis* and *C. elegans*, respectively) are represented by black dots. Transparent circles represent human elements. Their color code refers to the GPCR sub-families (AD: brown; AMIN: light blue; CHEM: dark blue; LGR: yellow; MEC: pink; MTN: grey; MRG: violet; OPN: orange; PEP: dark green; PTG: cyan; PUR: light green; SO: red; UC: black). The ellipses indicate the positions of the human G0 receptors.

It is worth noting that the evolution of orthologous sequences from the oldest ancestor common to an entire protein family can be decomposed into a shared part existing before speciation and a specific part originating after speciation. When sequences from one species are projected onto the sequence space of a reference species, this specific part is expected to be described by coordinates on high dimensions whereas the shared part should correspond to coordinates on the low dimension space of reference (i.e. to the position of the observed projected elements). This assumption is corroborated by the MDS analysis of GPCRs from the non-human sub-families present in *N. vectensis* and *C. elegans* whose projection onto the human space of reference overlaps group G0 (Figure 6).

### 3. Sequence determinants of GPCR evolution

**Figure 7:**
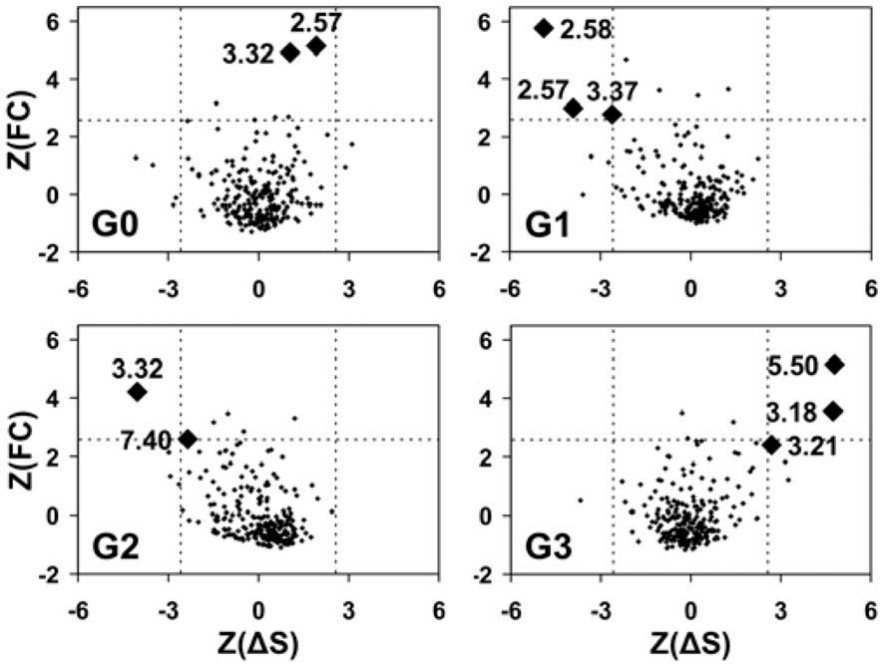
Sequence analysis of the four MDS groups. For each group *G_i_* (*i*  =  0 to 3) and each position *l* of the alignment, the *Z*-score of the correlation function, *FC*(*l*), is plotted as a function of the Z-score of the entropy difference Δ*S*(*l*) between group *G_i_* and its complement *G_i_^C^*. The dashed lines correspond to *Z*-scores of 2.58 (99% confidence level).

To search sequence determinants related to the evolutionary pathways observed by MDS, the aligned set of active and supplementary sequences was divided into four groups, according to the MDS classification of the human counterparts (Table 1). Positions specific of each MDS group (Figure 7) were searched for by plotting, for each position *l* of the MSA, the frequency correlation, *FC(l)*, as a function of the difference of entropy, *ΔS(l)* (see Methods). The position numbering is based on Ballesteros′ scheme [22]. The most conserved position in each transmembrane helix *n* (TM*n*) is numbered n.50 and is used as a relative reference.

A proline residue at position 2.58 in TM2 is the hallmark of G1 receptors. Present in SO receptors from *N. vectensis*, it is conserved in almost any G1 receptor [6]. The P2.58 pattern results from an indel (insertion/deletion) in TM2 [6] which appears as the key event yielding the emergence of this group. Recently, this indel received experimental validation with the resolution of the crystal structure of CXCR4 [23]. An aliphatic residue is also highly conserved at position 2.57 as a result of the indel. On the other hand, position 3.37 presents interesting characteristics. This position is variable in SO receptors from *N. vectensis* and *C. elegans* whereas it corresponds to Tyr in chordate SO and vertebrate CHEM and PUR receptors and to Phe in vertebrate SO receptors. This suggests that this position might be crucial for the evolution and the diversification of G1 receptors.

Two positions, 3.32 and 7.40, are specific of the AMIN receptors whose weight overwhelms AD receptors in G2. Interestingly, position 3.32 corresponds to an Asp residue in any species, whereas position 7.40 is a highly conserved Trp in any species except in *N. vectensis*, suggesting that this position is important in the evolution of AMIN receptors.

Three positions are highly specific of G3 receptors. However, these positions are *variable* in G3, whereas they are highly conserved in the other groups. The hallmark of G3 is the *absence* of P5.50 in TM5 which is frequently associated with the mutation of W3.18 and of G3.21 in the WXFG motif [24]. In addition, the proline residues in TM2 and TM5 are not independent (*p*-values < 10^-10^ with the χ^2^ test of independence) and the absence of proline in TM2 is also frequent in G3 (Table 1). It is interesting to note that the drift of PTG receptors along the second dimension is correlated with the partial loss of the TM2 proline in most recent species [6].

In contrast with the other groups, G0 does not possess hallmark residues. The positions with highest *FC*, 2.57 and 3.32, are only moderately conserved in G0 (28% Cys and 31% Gln, respectively) whereas they are highly conserved in G1 and G2, respectively. These positions, located within the extracellular side of the TM domain, face the receptor core and are ligand specific [25].

## Discussion

Introduced in the field of sequence analysis more than 20 years ago [26], mutidimensional scaling analysis was applied to the analysis of protein families [26], [27], [28], [29], [30], of the protein fold space [31], [32], [33], of virus evolution [34], [35], [36], [37] and of large genomic data sets [38]. This method usefully complements phylogenetic techniques and provides important insights into the evolution of proteins, genes and virus. In addition, compared to phylogenetic methods, MDS provides the possibility to project supplementary elements onto a reference space [15], [17], [18]. The projection of supplementary elements has been previously used with principal component analysis [20] and is also routinely used with correspondence analysis [39], [40]. However, to the best of our knowledge, the MDS projection technique has never been applied previously to the field of protein evolution. In this paper, we show that this technique provides invaluable information on the evolution of protein families that is not reachable by classical phylogenetic analysis.

In the MDS representation of the GPCR sequence space, receptors are clustered along a few privileged directions (Figure 2). Projection of receptors from supplementary species (Figure 4) helps interpret these directions in terms of evolutionary trends that are corroborated by sequence analysis (Figure 7). Several lines of evidence strongly suggest that the PEP sub-family forms a central node of GPCR evolution. First, its central position is maintained from cnidarians to vertebrates (Figure 4). Second, several sub-families (SO, AMIN, AD) are close to central PEP in the species most distantly related to humans, then they drift towards their position in the human space as the species are more closely related to humans (Figure 4–5). This is very striking for SO receptors whose vicinity to PEP receptors in non-chordate species corroborates our assumption of a common origin for these two sub-families [6]. Third, groups G1 to G3 are characterized by specific gain or loss of sequence patterns. This is not the case for group G0 (Figure 7). Fourth, the absence of proline in TM2 and/or TM5 is characteristic of “recent” sub-families, such as the MEC, PTG or MRG ones. This suggests that the LGR and OPN receptors may have evolved from an ancestor possessing proline residues in both helices whose PEP receptors might be the closest relative. This is consistent with the observation that substitutions from proline are more easily accommodated than substitutions to proline [41], [42]. Concerning the OPN sub-family, it should be added that there is no evidence of evolutionary linkage between prokaryotic and eukaryotic rhodopsins whose retinal-based photosensory system results from convergent evolution [43].

**Figure 8:**
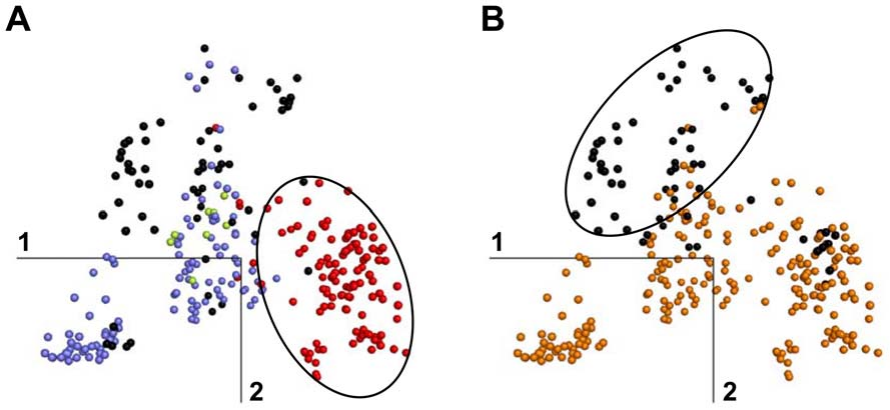
Proline patterns of human GPCRs. In (A), receptors with a proline residue at position 2.58, 2.59 or 2.60 in TM2 are red, slate or light green, respectively. Receptors with no proline in TM2 are black. The ellipse indicates G1 receptors. In (B), receptors with and without a proline residue at position 5.50 in TM5 are orange and black, respectively. The ellipse indicates G3 receptors.

Taken together, the MDS results support a model of radiative evolution of GPCRs from PEP receptors. In this model, the groups G1 to G3 defined by MDS correspond to three main evolutionary pathways from the ancestors of PEP receptors. The first evolutionary pathway was initiated by an indel in TM2, leading to the split of P2.59 PEP and P2.58 SO receptors [6]. The present data support the existence of a deletion mechanism that arose very early in GPCR evolution since receptors that can be assigned to the SO sub-family are present in cnidarians. The P2.58 proline pattern is the hallmark of this pathway (Figure 7 and 8A). However, the species drift of the SO sub-family indicates that the differentiation of SO from PEP receptors was progressive. It involved further mutations (e.g. at position 3.37) and eventually led to the vertebrate SO, CHEM and PUR sub-families by divergence (Figure 4). The second pathway is related to the differentiation of the AMIN receptors, characterized by the D3.32 pattern (Figure 7). Their drift (Figure 4–5) is partial in cnidarians, in agreement with the W7.40 sequence marker. AD receptors are part of this pathway, either by divergence from AMIN receptors or by convergence from PEP receptors.

The hallmark of the third evolutionary pathway is the mutation of proline residues in TM2 and/or TM5 (Figure 8), which is correlated with the mutation of the WXFG motif. However, the detailed analysis of these patterns (Table 1) does not indicate a unique mechanism. The PTG and MRG sub-families provide an example of reverse order in the mutation of the TM2 and TM5 proline residues. The split of the AD and MEC sub-families, related to the mutation of both proline residues in MEC receptors, is subsequent to the mutation of the WXFG motif in AD receptors. These data suggest that the sub-families from group G3 underwent parallel evolution in relation with a covarion process [9] in which the mutation of one of these sequence motifs releases structural and/or functional constraints and makes easier the subsequent mutation of the other motifs.

The mechanism of radiative evolution that we propose is consistent with the evolutionary trees obtained by neighbor-joining (NJ) or maximum parsimony (MP) methods for human and non-human species (dog, rat, pufferfish) that display a fan shape with sub-families from G1 on one hand and the AMIN sub-family on the other hand [6], [44], [45], [46]. In particular, this model is supported by the full consensus tree for rat and human GPCRs obtained from both NJ and MP analysis in which the position of the OPN, MRG, PTG and LGR sub-families is ambiguous [44]. It should be added that a classification of human GPCRs into four groups by MP [2] has enlightened the specificity of PEP receptors as a group. The discrepancy observed for the other groups might be explained by biases due to long branch attraction and/or to parallel evolution [12], [13].

It is worth noting that two of the main pathways of GPCR diversification are related to proline residues in transmembrane helices (Figure 8). Proline residues induce helical distortions that are key elements of GPCR structure and mechanism of activation. In particular, structural divergence between receptors may relate to the presence of proline [47], [48] whereas a seesaw motion of TM6, at the level of a highly conserved proline, is a crucial step of rhodopsin and β2 adrenoceptor activation [49], [50], [51]. We have previously proposed that the deletion in TM2 characteristic of G1 receptors modifies the distortion of this helix from a bulge to a “typical” proline kink [6]. This structural change is now experimentally validated [23]. How it affects the activation mechanism of G1 receptors remains to be determined. However, it is interesting to note that a rotational motion of TM2 upon activation, reminiscent of TM6, has been observed in the type I angiotensin II receptor which belongs to group G1 [52].

Along with the TM2 proline, the TM5 proline appears as a major vector of GPCR evolution. The correlation of the TM2 and TM5 proline mutations observed in independent sub-families of group G3 is indicative of a covarion process. Comparison of the active and inactive structures of rhodopsin [48], [49] and of the β2 adrenoceptor [47], [50] provides some hints for this long range effect (25 Å). In either case, the inactive sate is stabilized by interactions involving TM3 with both TM5 through P5.50 and TM2 through its bulge at position 2.56 or 2.57. In the active state, however, these interactions are impaired. The mutation of either proline should thus affect the stability of the inactive state, either directly (TM5) or through the structure of the related bulge (TM2). The correlation of these mutations in G3 sub-families suggests that a similar reorganization of the interaction network stabilizing the inactive state might be shared by G3 receptors.

In conclusion, MDS is especially suited for the analysis of large and diversified protein families, such as GPCRs, whose phylogenetic relationships between numerous sub-families are unclear. In the case of GPCRs, it emphasizes the usefulness of rare mutational events, such as indels or mutations of residues with strong structural and/or functional constraints, to infer the evolution of protein families. In addition, the projection of supplementary sequences onto a sequence space of reference is an important tool to compare orthologous sequences. As exemplified with GPCRs, the MDS projection technique allows a straightforward and spectacular visualization of the evolutionary drift of different sub-families. It helps decipher hallmark and lineage-specific mutational events that drove sub-family evolution, and provides insights into the mechanisms that led to the molecular diversification of a protein family.

## Methods

### Sequences of class A GPCRs

The non-redundants sets of non-olfactory class A GPCRs from *C. elegans*, *C. intestinalis*, *D. rerio* and *H. sapiens* (109, 90, 236 and 283 sequences, respectively) correspond to the previously determined sets [6], updated with the July 2009 release of Uniprot when necessary. 93% of the human receptors can be assigned to twelve sub-families (Table 1), whereas 7% of them remain unclassified (UC). The sub-family nomenclature is adapted from Fredriksson′s classification [2]. The ratio of sequences assigned to these twelve sub-families is 57, 87, and 95% for *C. elegans*, *C. intestinalis,* and *D. rerio*, respectively. The sequence set of class A GPCRs from *N. vectensis* was prepared from the July 2009 release of Uniprot, according to the procedure previously described [6]. It is composed of 538 non-redundant (identity < 90%), non-olfactory sequences, 26% of which could be assigned to GPCR sub-families present in humans. The remaining sequences belong to GPCR sub-families specific of cnidarians [53]. The accession numbers of the sequences used for this study are given in Data S1.

Multiple sequence alignments were carried out with ClustalX [54] and manually refined with GeneDoc [55] to insure that the anchor residue of each helix was correctly aligned. The anchor residues corresponding to the most conserved positions are N1.50, D2.50, R3.50, W4.50, P5.50, P6.50 and P7.50 (Ballesteros′ numbering [22]). For the less conserved TM5, we used either P5.50 or Y5.58 to insure correct alignment. Sequence analyses were carried out on the MSA positions with less than 2% gaps. These 236 positions correspond to residues 1.30–1.62, 2.37–2.65, 3.18–3.59, 4.37–4.63, 5.34–5.65, 6.24–6.61 and 7.30–7.64. They include the seven transmembrane helices, the putative eighth intracellular helix and parts of the intracellular and extracellular loops.

A random multiple sequence alignment was built from 283 random sequences of 236 amino acids and was used as a control for the MDS analysis of human GPCRs.

### Multidimensional scaling analysis

When a set of sequences (referred to as active sequences) are aligned, a distance between each pair of sequences can be calculated from the MSA. The matrix of the pairwise distances can then be analyzed by MDS [15], [16]. Formally, if we denote by *N* the number of sequences, by **D** the *N* by *N* the matrix of the squared distance between sequences, by **I** the *N* by *N* identity matrix, and by **1** an *N* by *N* matrix of ones, the first step is to transform the distance matrix **D** into a cross-product matrix denoted **S** and computed as:
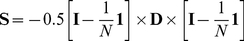
(1)The eigendecomposition of **S** expresses this matrix as the diagonal matrix of the eigenvalues **Λ** multiplied on the left and on the right by the eigenvector matrix **U** (such as **S**  =  **UΛU** ^T^, where ^T^ denotes the transposition operation). The eigenvectors of **S**, or principal components, form the active space. The factor score matrix, denoted **F**, is computed as:
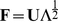
(2)and gives the coordinates of the active elements in the active space.

Additional sequences are projected onto the active space as supplementary elements [15], according to the procedure summarized in Figure 1. First, supplementary sequences are aligned against the active MSA, resulting into a supplementary matrix of distances between the supplementary and active sequences. Then, the supplementary distance matrix is transformed into a supplementary cross-product matrix which is in turn transformed into a factor matrix (Figure 1). Specifically, if we denote *N*_sup_ the number of supplementary sequences, **1**_sup_ an *N*_sup_ by *N* matrix of ones, and **D**_sup_ the supplementary squared distance matrix, then the first step is to transform **D**_sup_ into a cross product matrix denoted **S**_sup_ as:
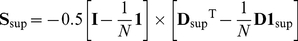
(3)The factor matrix for the supplementary sequences, denoted **F**_sup_, is computed as:
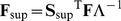
(4)and gives the coordinates of the supplementary elements in the active space.

The simplest pairwise distance is given by the proportion of sites that differ between the two sequences [56]. It yields a distance very close to an Euclidian distance, because the eigendecomposition of the matrix based on this distance gives a small proportion of negative eigenvalues representing only 3% of the sum of absolute eigenvalues. Distances based on generic or transmembrane specific scoring matrices [57] do not perform as well, as indicated by the fact that their negative eigenvalues represent from 4 to 10% of the sum. Pairwise or complete deletion of gap positions does not yield significant differences in the results because of the small amount of gaps in the MSA (only positions with less than 2% gaps were considered). The data shown are obtained with distances based on sequence identity and pairwise deletion of gaps.

### Receptor clustering

Following MDS, receptors were mapped in a 3D space and clustered by *K*-means analysis. The *K*-means procedure was reiterated 1000 times with random initial centroids. The most frequent clustering, which was in agreement with visual inspection, was selected and used as a reference to assess the reproducibility of the analysis. More than 97% of the receptors were assigned to the same reference cluster in more than 85% of the runs. The Silhouette score [19] was calculated from *K*-means clustering with the number of clusters ranging from 1 to 13 (for the 12 sub-families and UC receptors). For each number of clusters, 1000 runs were averaged.

### Sequence analysis

When a sequence set is divided into a subset g and its complement *g^C^*, the correlation between a position *l* of the MSA and the subsets is measured by the frequency correlation *FC(l)*, derived from the χ^2^ test [58], according to the formula: 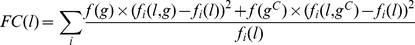
(5)where *f(g)* and *f(g^C^*) are the frequencies of *g* and *g^C^*, respectively, and *f_i_(l)*, *f_i_(l,g)* and *f_i_(l,g^C^*) are the frequencies of amino acid i at position *l* in the entire set, in g and in *g^C^*, respectively. *FC(l)* varies from 0 for totally variable positions to 1 for positions fully correlated with the subsets. In addition, the difference of sequence entropy [59] between *g* and *g^C^* is given by:
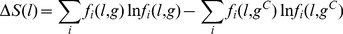
(6)Specific conservation or variability in the subset g corresponds to negative and positive values of Δ*S*, respectively. Sequence determinants of g are searched for by plotting the *Z*-scores of *FC(l)* as a function of the *Z*-scores of Δ*S(l)*.

### Figure preparation

The MDS figures were prepared with the PyMOL molecular graphics system [60], after formatting the MDS coordinates on the first three dimensions as a Protein Data Bank file.

## Supporting Information

Data S1 Accession numbers of the GPCR sequences used to build the multiple sequence alignments analyzed by MDS.
(PDF)

## References

[1] Gether U (2000). Uncovering molecular mechanisms involved in activation of G protein-coupled receptors.. Endocr Rev.

[2] Fredriksson R, Lagerstrom MC, Lundin LG, Schioth HB (2003). The G-protein-coupled receptors in the human genome form five main families. Phylogenetic analysis, paralogon groups, and fingerprints.. Mol Pharmacol.

[3] Kolakowski LF (1994). GCRDb: a G-protein-coupled receptor database.. Receptors Channels.

[4] Surgand JS, Rodrigo J, Kellenberger E, Rognan D (2006). A chemogenomic analysis of the transmembrane binding cavity of human G-protein-coupled receptors.. Proteins.

[5] Vassilatis DK, Hohmann JG, Zeng H, Li F, Ranchalis JE (2003). The G protein-coupled receptor repertoires of human and mouse.. Proc Natl Acad Sci U S A.

[6] Devillé J, Rey J, Chabbert M (2009). An indel in transmembrane helix 2 helps to trace the molecular evolution of class A G-protein-coupled receptors.. J Mol Evol.

[7] Rokas A, Carroll SB (2006). Bushes in the tree of life.. PLoS Biol.

[8] Rokas A, Kruger D, Carroll SB (2005). Animal evolution and the molecular signature of radiations compressed in time.. Science.

[9] Fitch WM (1971). Rate of change of concomitantly variable codons.. J Mol Evol.

[10] Studer RA, Robinson-Rechavi M (2010). Large-scale analysis of orthologs and paralogs under covarion-like and constant-but-different models of amino acid evolution.. Mol Biol Evol.

[11] Tuffley C, Steel M (1998). Modeling the covarion hypothesis of nucleotide substitution.. Math Biosci.

[12] Susko E, Inagaki Y, Roger AJ (2004). On inconsistency of the neighbor-joining, least squares, and minimum evolution estimation when substitution processes are incorrectly modeled.. Mol Biol Evol.

[13] Wang HC, Susko E, Spencer M, Roger AJ (2008). Topological estimation biases with covarion evolution.. J Mol Evol.

[14] Togerson WS (1958). Theory and methods of scaling..

[15] Abdi H, Salkind NJ (2007). Metric multidimensional scaling.. *Encyclopedia of Measurement and Statistics*.

[16] Takane Y, Jung S, Oshima-Takane Y, Millsap R, Maydeu-Olivares A (2009). Multidimensional scaling.. Handbook of quantitative methods in psychology.

[17] Gower JC (1968). Adding a Point to Vector Diagrams in Multivaraiate Analysis Biometrika.

[18] Trosset MW, Pribe CE (2008). The out-of-sample problem for classical multidimensional scaling.. Computational Statistics & Data Analysis.

[19] Rousseeuw P (1987). Silhouettes: A Graphical Aid to the Interpretation and Validation of Cluster Analysis.. J Comput Appl Math.

[20] Abdi H, Williams LJ (2010). Principal component analysis.. Wiley Interdisciplinary reviews: Computational Statistics.

[21] Fredriksson R, Schioth HB (2005). The repertoire of G-protein-coupled receptors in fully sequenced genomes.. Mol Pharmacol.

[22] Sealfon SC, Chi L, Ebersole BJ, Rodic V, Zhang D (1995). Related contribution of specific helix 2 and 7 residues to conformational activation of the serotonin 5-HT2A receptor.. J Biol Chem.

[23] Wu B, Chien EY, Mol CD, Fenalti G, Liu W (2010). Structures of the CXCR4 chemokine GPCR with small-molecule and cyclic peptide antagonists.. Science.

[24] Peeters MC, van Westen GJ, Li Q, Ijzerman AP (2011). Importance of the extracellular loops in G protein-coupled receptors for ligand recognition and receptor activation.. Trends Pharmacol Sci.

[25] Ye K, Lameijer EW, Beukers MW, Ijzerman AP (2006). A two-entropies analysis to identify functional positions in the transmembrane region of class A G protein-coupled receptors.. Proteins.

[26] Woolley KJ, Athalye M (1986). A use for principal coordinate analysis in the comparison of protein sequences.. Biochem Biophys Res Commun.

[27] Higgins DG (1992). Sequence ordinations: a multivariate analysis approach to analysing large sequence data sets.. Comput Appl Biosci.

[28] Casari G, Sander C, Valencia A (1995). A method to predict functional residues in proteins.. Nat Struct Biol.

[29] Gogos A, Jantz D, Senturker S, Richardson D, Dizdaroglu M (2000). Assignment of enzyme substrate specificity by principal component analysis of aligned protein sequences: an experimental test using DNA glycosylase homologs.. Proteins.

[30] Lu F, Keles S, Wright SJ, Wahba G (2005). Framework for kernel regularization with application to protein clustering.. Proc Natl Acad Sci U S A.

[31] Hou J, Jun SR, Zhang C, Kim SH (2005). Global mapping of the protein structure space and application in structure-based inference of protein function.. Proc Natl Acad Sci U S A.

[32] Hou J, Sims GE, Zhang C, Kim SH (2003). A global representation of the protein fold space.. Proc Natl Acad Sci U S A.

[33] Choi IG, Kim SH (2006). Evolution of protein structural classes and protein sequence families.. Proc Natl Acad Sci U S A.

[34] Blackshields G, Sievers F, Shi W, Wilm A, Higgins DG (2010). Sequence embedding for fast construction of guide trees for multiple sequence alignment.. Algorithms Mol Biol.

[35] Kuiken C, Hraber P, Thurmond J, Yusim K (2008). The hepatitis C sequence database in Los Alamos.. Nucleic Acids Res.

[36] Shi W, Lei F, Zhu C, Sievers F, Higgins DG (2010). A complete analysis of HA and NA genes of influenza A viruses.. PLoS One.

[37] Smith DJ, Lapedes AS, de Jong JC, Bestebroer TM, Rimmelzwaan GF (2004). Mapping the antigenic and genetic evolution of influenza virus.. Science.

[38] Tzeng J, Lu HH, Li WH (2008). Multidimensional scaling for large genomic data sets.. BMC Bioinformatics.

[39] Greenacre M (2007). Correspondance analysis in practice..

[40] Murtagh F (2005). Correspondence Analysis and data Coding with R and Java..

[41] Yohannan S, Faham S, Yang D, Whitelegge JP, Bowie JU (2004). The evolution of transmembrane helix kinks and the structural diversity of G protein-coupled receptors.. Proc Natl Acad Sci U S A.

[42] Yohannan S, Yang D, Faham S, Boulting G, Whitelegge J (2004). Proline substitutions are not easily accommodated in a membrane protein.. J Mol Biol.

[43] Rompler H, Staubert C, Thor D, Schulz A, Hofreiter M (2007). G protein-coupled time travel: evolutionary aspects of GPCR research.. Mol Interv.

[44] Gloriam DE, Fredriksson R, Schioth HB (2007). The G protein-coupled receptor subset of the rat genome.. BMC Genomics.

[45] Haitina T, Fredriksson R, Foord SM, Schioth HB, Gloriam DE (2009). The G protein-coupled receptor subset of the dog genome is more similar to that in humans than rodents.. BMC Genomics.

[46] Metpally RP, Sowdhamini R (2005). Genome wide survey of G protein-coupled receptors in Tetraodon nigroviridis.. BMC Evol Biol.

[47] Cherezov V, Rosenbaum DM, Hanson MA, Rasmussen SG, Thian FS (2007). High-resolution crystal structure of an engineered human beta2-adrenergic G protein-coupled receptor.. Science.

[48] Palczewski K, Kumasaka T, Hori T, Behnke CA, Motoshima H (2000). Crystal structure of rhodopsin: A G protein-coupled receptor.. Science.

[49] Park JH, Scheerer P, Hofmann KP, Choe HW, Ernst OP (2008). Crystal structure of the ligand-free G-protein-coupled receptor opsin.. Nature.

[50] Rasmussen SG, Choi HJ, Fung JJ, Pardon E, Casarosa P (2011). Structure of a nanobody-stabilized active state of the beta(2) adrenoceptor.. Nature.

[51] Scheerer P, Park JH, Hildebrand PW, Kim YJ, Krauss N (2008). Crystal structure of opsin in its G-protein-interacting conformation.. Nature.

[52] Domazet I, Holleran BJ, Martin SS, Lavigne P, Leduc R (2009). The second transmembrane domain of the human type 1 angiotensin II receptor participates in the formation of the ligand binding pocket and undergoes integral pivoting movement during the process of receptor activation.. J Biol Chem.

[53] Anctil M, Hayward DC, Miller DJ, Ball EE (2007). Sequence and expression of four coral G protein-coupled receptors distinct from all classifiable members of the rhodopsin family.. Gene.

[54] Larkin MA, Blackshields G, Brown NP, Chenna R, McGettigan PA (2007). Clustal W and Clustal X version 2.0.. Bioinformatics.

[55] Nicholas KB, NHB, Deerfield DWI (1997). GeneDoc: Analysis and Visualization of Genetic Variation.. EMBNEW NEWS.

[56] Nei M, Zhang J (2005). Evolutionary Distance: Estimation..

[57] Grishin VN, Grishin NV (2002). Euclidian space and grouping of biological objects.. Bioinformatics.

[58] Kass I, Horovitz A (2002). Mapping pathways of allosteric communication in GroEL by analysis of correlated mutations.. Proteins.

[59] Valdar WS (2002). Scoring residue conservation.. Proteins.

[60] DeLano JW (2002). http://www.pymol.org.

