# Supplementary material for: Multidimensional Scaling Reveals the Main Evolutionary Pathways of Class A G-Protein-Coupled Receptors
Source: PLoS One. 2011 Apr 22;6(4):e19094. doi: 10.1371/journal.pone.0019094 (PMC3081337; doi:10.1371/journal.pone.0019094)
Supplement: Data S1 — Accession numbers of the GPCR sequences used to build the multiple sequence alignments analyzed by MDS. (PDF) [file pone.0019094.s001.pdf]

Data S1

Accession numbers of the GPCR sequences used to build the multiple sequence alignments analyzed by MDS

The accession numbers correspond to the UniProtKB accession numbers except for the sequences from *C. Intestinalis* with references CI0xxxxxx. They correspond to the sequences reported by Fredrikkson et al. (2005) under the references lcl|ci0100xxxxxx.

H. sapiens

O00155 O00254 O00270 O00398 O00421 O00574 O00590 O14626 O14718 O14804
O14842 O14843 O15218 O15354 O15552 O43193 O43194 O43603 O43613 O43614
O60755 O60883 O75388 O75473 O95136 O95665 O95800 O95977 P03999 P04000
P04201 P07550 P08100 P08172 P08173 P08588 P08908 P08912 P08913 P11229
P13945 P14416 P16473 P18089 P18825 P20309 P21452 P21453 P21462 P21554
P21728 P21730 P21731 P21917 P21918 P22888 P23945 P24530 P25021 P25024
P25025 P25089 P25090 P25100 P25101 P25103 P25105 P25106 P25116 P25929
P28221 P28222 P28223 P28335 P28336 P28566 P29274 P29275 P29371 P30411
P30518 P30542 P30550 P30556 P30559 P30872 P30874 P30939 P30968 P30989
P31391 P32238 P32239 P32245 P32246 P32247 P32248 P32249 P32302 P32745
P33032 P33765 P34969 P34972 P34981 P34995 P35346 P35348 P35367 P35368
P35372 P35408 P35410 P35414 P35462 P37288 P41143 P41145 P41146 P41231
P41595 P41597 P41968 P43088 P43115 P43116 P43119 P43657 P46089 P46091
P46092 P46093 P46094 P46095 P46663 P47211 P47775 P47804 P47898 P47900
P47901 P48039 P48145 P48146 P49146 P49238 P49286 P49682 P49683 P49685
P50052 P50391 P50406 P51582 P51677 P51679 P51681 P51684 P51685 P51686
P55085 P60893 P61073 Q01718 Q01726 Q13258 Q13304 Q13585 Q13639 Q14330
Q14439 Q15077 Q15391 Q15722 Q15743 Q15760 Q15761 Q16581 Q49SQ1 Q5KU14
Q5KU21 Q5NUL3 Q6DWJ6 Q6U736 Q6W5P4 Q7Z601 Q7Z602 Q86SM5 Q86SM8 Q86VZ1
Q8IYL9 Q8IZ08 Q8N6U8 Q8NDV2 Q8NFJ6 Q8NGU9 Q8TCW9 Q8TDS4 Q8TDS5 Q8TDS7
Q8TDT2 Q8TDU6 Q8TDU9 Q8TDV0 Q8TDV2 Q8TDV5 Q8WXD0 Q92633 Q92847 Q969F8
Q969N4 Q969V1 Q96AM1 Q96CH1 Q96G91 Q96LA9 Q96LB0 Q96LB1 Q96LB2 Q96P65
Q96P66 Q96P67 Q96P68 Q96P69 Q96P88 Q96RI0 Q96RI8 Q96RI9 Q96RJ0 Q99500
Q99527 Q99677 Q99678 Q99679 Q99680 Q99705 Q99788 Q9BPV8 Q9BXA5 Q9BXB1
Q9BXC0 Q9BXC1 Q9BY21 Q9BZJ6 Q9BZJ7 Q9BZJ8 Q9GZN0 Q9GZQ4 Q9GZQ6 Q9H1C0
Q9H1Y3 Q9H228 Q9H244 Q9H3N8 Q9HB89 Q9HBW0 Q9HBX8 Q9HBX9 Q9HC97 Q9NPB9
Q9NPC1 Q9NQS5 Q9NS66 Q9NS67 Q9NS75 Q9NSD7 Q9NYM4 Q9P1P4 Q9P1P5 Q9P296
Q9UBY5 Q9UHM6 Q9UJ42 Q9UKP6 Q9UNW8 Q9UPC5 Q9Y271 Q9Y2T5 Q9Y2T6 Q9Y5N1
Q9Y5X5 Q9Y5Y3 Q9Y5Y4

D. rerio

A0PJP9 A0PJR8 A0PJS0 A0T2N3 A1A5V5 A2AR72 A2AV71 A2AVM2 A2BG57 A2BGL4
A2BGL6 A2BGT9 A2BHH9 A2BIP4 A2BIP6 A2CEA3 A3KP34 A3KP39 A3KPR9 A3KPY3
A3KQC8 A3QJX2 A3QJX7 A3QJZ0 A3QK20 A4FUN8 A4QNU5 A4QNV7 A5PF47 A5PLC2
A5PLE7 A5PMA5 A5PMT9 A5WUE9 A5WUN1 A5WW93 A7E2M1 A7KBS6 A7MBV0 A7UD89
A8CAH3 A8CAH4 A8DZ94 A8E4T2 A8E7L5 A8E7S9 A8E7T8 A8KB27 A8KBU0 A8WGB2
A8YXX6 A9JRC2 A9JRH6 A9JRY2 A9JRY7 A9NJI7 B0R0R2 B0R162 B0R167 B0S4Q6
B0S4Y3 B0S588 B0S5B5 B0S5K3 B0S6G9 B0S6Q0 B0S6X1 B0S732 B0UXR0 B0UYT9
B0V116 B0V1I9 B0V1K7 B0V2S3 B1NYI1 B1NYI2 B1NYI3 B2WSB2 B3DGM7 B3DGN0
B3DH72 B3DHI5 B3DHM5 B3DHM6 B3DIE4 B3DIJ2 B3DJ81 B3DJ99 B3DJA3 B3DJU7
B3DJU8 B3DK10 B3DK14 B3DK58 B3DKN8 B3DLG5 B3G515 B6E506 O57463 O73734
P0C7U4 P0C7U5 P35359 P51046 P51049 Q0GH74 Q195J0 Q195J2 Q1JPS6 Q1L955
Q1L962 Q1LUG8 Q1LVD8 Q1LVV2 Q1LX14 Q1LXU7 Q1LY72 Q1LYI4 Q1MT85 Q1RLT8
Q29ST4 Q29ST5 Q29ST6 Q2KNE5 Q2MV23 Q32LU0 Q32PK6 Q4FE81 Q502K5 Q535D9
Q567Y2 Q5CZS2 Q5DJ14 Q5DJ15 Q5DJ16 Q5QNM9 Q5QNN0 Q5QNN1 Q5QNN2 Q5QNN3
Q5QNN7 Q5QNN8 Q5QNN9 Q5QNP0 Q5QNP2 Q5QNP3 Q5QNP5 Q5QNP6 Q5QNQ0 Q5QNQ1
Q5QNQ2 Q5QNQ3 Q5RFY4 Q5RFY5 Q5RFY6 Q5RG92 Q5RG93 Q5RG95 Q5RGL2 Q5RGR7
Q5RHQ9 Q5RIG7 Q5RIV6 Q5SPG6 Q5TYV5 Q5U387 Q5U389 Q5U3D9 Q5VJN2 Q5VJN3
Q5XJD9 Q66I55 Q6DC27 Q6DG12 Q6DGJ2 Q6DH22 Q6NV10 Q6PHE1 Q6PHK5 Q6PR57
Q6WZB3 Q6XCC5 Q6XCC6 Q6XCC7 Q6ZM33 Q7SZP9 Q7T1A1 Q7T1A2 Q7T298 Q7T3E9
Q7T3Q3 Q7T3Q4 Q7ZTA1 Q7ZTA2 Q7ZTA3 Q7ZZ32 Q7ZZ93 Q7ZZC0 Q801F5 Q801F6
Q8AWE0 Q8AYM8 Q8AYN0 Q8JFR3 Q8JG69 Q8JG70 Q8JGW1 Q8JGW2 Q8JGW3 Q8UUW8
Q90WY4 Q90WY5 Q90WY6 Q9DDK4 Q9DGM2 Q9I8K8 Q9I918 Q9I919 Q9IB88 Q9PTF7

Q9PTX9 Q9W6A6 Q9W6A8 Q9W6A9

C. intestinalis

CI0130320 CI0130612 CI0130986 CI0131140 CI0131758 CI0132133 CI0132380
CI0132620 CI0133186 CI0133550 CI0133606 CI0133821 CI0134145 CI0134273
CI0134424 CI0136041 CI0137355 CI0137803 CI0137823 CI0137935 CI0138509
CI0138913 CI0138955 CI0139176 CI0140881 CI0141702 CI0141745 CI0141751
CI0142029 CI0142069 CI0142353 CI0143219 CI0143330 CI0143362 CI0143568
CI0143701 CI0144199 CI0144368 CI0144713 CI0144874 CI0144925 CI0145358
CI0145437 CI0146266 CI0146328 CI0147271 CI0147407 CI0147431 CI0147526
CI0148288 CI0148400 CI0148454 CI0148475 CI0149551 CI0150116 CI0150689
CI0150840 CI0151225 CI0151424 CI0151600 CI0153351 CI0153785 CI0153810
CI0153844 CI0154135 CI0154530 Q14SS9 Q3ZK33 Q3ZK34 Q3ZK35
Q3ZK36 Q60GS8 Q69HP5 Q69HR2 Q69HS9 Q70SX9 Q8IU30
Q95P33

C. elegans

Human sub-families
O02213 O02300 O17239 O18014 O18512 O44148 O44536 O44731 O44986 O45725
O45732 O62059 O62062 O62169 O62189 O62403 P34311 P90745 P90927 P91439
Q03566 Q03613 Q09388 Q09502 Q09638 Q18007 Q18179 Q18534 Q18701 Q18759
Q18775 Q18904 Q19084 Q1ZXT1 Q20067 Q20275 Q20325 Q20841 Q22188 Q23013
Q23033 Q23305 Q23497 Q2V4S5 Q58AU1 Q59E84 Q6EUU1 Q6RYS9 Q7JNX1 Q7JP62
Q7KPZ5 Q7YXG8 Q86GT6 Q86ME4 Q8MXS7 Q95YD7 Q9GZG8 Q9N324 Q9N4R5 Q9U7D5
Q9XTF5 Q9XUK8

Non-human sub-families
Q19399 Q19999 Q18659 Q94219 Q21413 O45613 Q93704 Q9N5A3 O45174 Q9GYH3
Q19397 O16548 Q17478 Q18321 O45601 Q17594 YWO1 YMJC Q21957 Q95QA0
Q23265 Q8MQ19 Q8MQF9 Q22485 Q17553 Q9XVS5 O62168 Q3S1L7 Q8I7K6 Q18228
Q22305 Q9N476 YT66 Q18876 Q18816 Q9U320 Q18923 O45173 Q9GYH2 Q18929
O02043 O17029 O45096 Q20715 Q20101 O17899 O16268

N. vectensis

Human sub-families
A7RF33 A7RF67 A7RF83 A7RFV1 A7RGG2 A7RI24 A7RIF2 A7RIJ2 A7RIJ3 A7RJ64
A7RJ85 A7RJ86 A7RJ90 A7RLJ1 A7RLJ5 A7RMI7 A7RMJ9 A7RN36 A7RN95 A7RNA2
A7RNS8 A7RNV5 A7RR06 A7RR08 A7RR17 A7RRU2 A7RS86 A7RS95 A7RS96 A7RSG0
A7RSL9 A7RSM8 A7RTL7 A7RTT1 A7RU09 A7RVG9 A7RVQ0 A7RVS6 A7RW88 A7RWI5
A7RWW9 A7RXA6 A7RXB4 A7RXF4 A7RYI8 A7RYJ5 A7RYX0 A7RZ25 A7RZ84 A7RZD5
A7RZL7 A7RZN0 A7RZT8 A7S0P3 A7S178 A7S190 A7S191 A7S1B8 A7S1R6 A7S1R7
A7S367 A7S3P4 A7S3S6 A7S4C0 A7S4M1 A7S4X4 A7S552 A7S585 A7S5U1 A7S7P5
A7S855 A7S856 A7S8Q1 A7S8Q5 A7S8R9 A7S8U4 A7S8Z0 A7S953 A7S9X3 A7SA66
A7SB78 A7SBY6 A7SC98 A7SDC0 A7SFC6 A7SG00 A7SGB7 A7SGW8 A7SH45 A7SHS8
A7SI85 A7SIA0 A7SIA3 A7SIH8 A7SIH9 A7SIX0 A7SJL3 A7SK22 A7SKS2 A7SL25
A7SLG6 A7SM59 A7SN38 A7SNN3 A7SPG8 A7SR60 A7SRR1 A7SRV7 A7SSB7 A7ST92
A7STC6 A7STN0 A7SU96 A7SUF5 A7SUG2 A7SW40 A7SWM7 A7SX62 A7SY03 A7SYQ1
A7SZI5 A7T0F7 A7T171 A7T2P7 A7T607 A7T7U0 A8DVC5 A9UMX1 A9UMX2 A9UMX3
A9UMX5 A9UMX6 A9UMX7 A9UMX9 A9UMY0 A9UMY4 A9UMY8 A9UMY9 A9UMZ2 A9UMZ3
A9UMZ5

Non-human sub-families
A7RIA3 A7RX52 A7SEY4 A7SUW4 A7SIK3 A7SDN2 A7RK50 A7RKF1 A7S0V6 A7SGT8
A7RIH7 A7RX80 A7SEZ1 A7SV87 A7SIK4 A7SEA3 A7RKH8 A7RKZ2 A7S158 A7SH26
A7RJ24 A7RYK8 A7SF51 A7SVL0 A7SN28 A7SH32 A7RKH9 A7RLK8 A7S1V6 A7SH27
A7RJ58 A7RZ31 A7SF75 A7SVR3 A7SPE4 A7SH33 A7RKS1 A7RM14 A7S1V7 A7SHH2
A7RJ65 A7RZ39 A7SF78 A7SWN7 A7SSI2 A7SI72 A7RQ52 A7RM19 A7S259 A7SI17
A7RJD4 A7RZ43 A7SFG7 A7SWQ4 A7SSZ5 A7SKC4 A7RQ59 A7RMP6 A7S2M6 A7SI22
A7RJD5 A7RZD0 A7SGX5 A7SX59 A7SYI7 A7SKF4 A7RQE1 A7RMT7 A7S3H8 A7SIX7
A7RKE1 A7RZE3 A7SGY4 A7SZG5 A7SYQ9 A7SKI1 A7RQG0 A7RMY3 A7S3I8 A7SJ28
A7RKE6 A7RZJ2 A7SHI6 A7SZU5 A7T1W6 A7SMC5 A7RQH6 A7RP54 A7S3W6 A7SJ90
A7RKX4 A7RZT0 A7SHP0 A7T159 A7T2K5 A7SN95 A7RU26 A7RPL7 A7S4R6 A7SJ99
A7RM98 A7S074 A7SHV9 A7T202 A7TDI1 A7SS08 A7RUG9 A7RPM6 A7S522 A7SJA6
A7RMD8 A7S1A7 A7SI80 A7T260 A7REP3 A7STD9 A7RY66 A7RQC6 A7S6D4 A7SKN7
A7RMF3 A7S1K2 A7SIR1 A7T8T2 A7RES1 A7STL2 A7RYR4 A7RQP0 A7S6M9 A7SL32
A7RMX9 A7S2E1 A7SJN7 A7TCJ5 A7REU0 A7SU66 A7S495 A7RQR1 A7S738 A7SLK3

A7RN94 A7S2I9 A7SJS8 A8DUY3 A7RKK1 A7SUE1 A7S8R2 A7RQX6 A7S7C9 A7SMC7
A7RNT4 A7S2T9 A7SJS9 A7RFM1 A7RKN5 A7SV24 A7SBP0 A7RR28 A7S7Y4 A7SMF9
A7RQ15 A7S2W3 A7SJT1 A7RGV2 A7RP65 A7SV82 A7SCM1 A7RRX3 A7S8C6 A7SMG9
A7RQS9 A7S343 A7SJT6 A7RJC8 A7RPQ9 A7T0L0 A7SDJ2 A7RS06 A7S974 A7SMM8
A7RQT5 A7S4A0 A7SJT7 A7RKP0 A7RQT2 A7T4G9 A7SIZ4 A7RS46 A7S9K3 A7SNV7
A7RQX8 A7S595 A7SJT8 A7RKW8 A7RRJ3 A7RHI8 A7SIZ6 A7RSF8 A7S9Y9 A7SP63
A7RR71 A7S5B7 A7SK68 A7RRH0 A7RRX7 A7RQ78 A7SLE0 A7RT84 A7SA37 A7SQW8
A7RRB2 A7S5H8 A7SKD4 A7RRJ1 A7RTI9 A7RXC3 A7SMQ7 A7RTV0 A7SB08 A7SRC7
A7RRN3 A7S651 A7SKD5 A7RSP6 A7RTJ2 A7S1I8 A7SPU9 A7RTV5 A7SBC3 A7SSU5
A7RRT8 A7S6A7 A7SKL7 A7RSS6 A7RU74 A7S1R1 A7SVX2 A7RVA6 A7SBI5 A7SSY9
A7RRW1 A7S737 A7SKY5 A7RXY5 A7RV10 A7S551 A7SWJ2 A7RVC6 A7SBJ7 A7SVI7
A7RS56 A7S768 A7SLE8 A7RYQ3 A7RV11 A7S662 A7T1I9 A7RW09 A7SCA7 A7SVM9
A7RSG5 A7S7I1 A7SM13 A7RYZ7 A7RV12 A7S9Z8 A7T2F5 A7RW16 A7SD06 A7SVN0
A7RSR4 A7S7W2 A7SMD8 A7S169 A7RVU9 A7S9Z9 A7T4L9 A7RW36 A7SD07 A7SXR6
A7RTB1 A7S987 A7SMD9 A7S581 A7RW35 A7SAD6 A7RET5 A7RX53 A7SD63 A7SXR7
A7RTH4 A7SAD3 A7SMQ6 A7S5N6 A7RWC2 A7SF36 A7RET9 A7RX85 A7SDE2 A7SY91
A7RU07 A7SB18 A7SPC3 A7S8N0 A7RXI4 A7SLG5 A7RFK8 A7RXG0 A7SEV0 A7SYD2
A7RU57 A7SB82 A7SPE0 A7SAM1 A7RXX4 A7SLN8 A7RFP2 A7RXP9 A7SEV2 A7SYD4
A7RUB1 A7SBN8 A7SPH3 A7SAY6 A7RY07 A7SLS4 A7RFU8 A7RXU3 A7SF45 A7SYU2
A7RUG4 A7SBW7 A7SPL5 A7SAY7 A7RZ77 A7SLS6 A7RG34 A7RXX8 A7SF59 A7SZG6
A7RUT9 A7SC55 A7SSA5 A7SAY8 A7S1Q8 A7SR88 A7RHQ2 A7RYJ0 A7SFD2 A7T1W3
A7RVH5 A7SCR3 A7SSQ1 A7SAY9 A7S376 A7REP5 A7RIZ4 A7RYQ5 A7SFI1 A7T1X9
A7RVV4 A7SD52 A7SSZ2 A7SB34 A7S497 A7RF92 A7RJ80 A7RZA3 A7SFL8 A7T1Y0
A7RVZ2 A7SD89 A7STR1 A7SFM3 A7S7N3 A7RHR7 A7RJD3 A7RZJ1 A7SG21 A7T1Y2
A7RW17 A7SDA9 A7STR3 A7SGJ8 A7S7Q4 A7RJG8 A7RJF2 A7S0P4 A7SGL8 A7T361
A7RWH6 A7SDD1 A7SUU8 A7SHN4 A7SB28 A7RJW0 A7RKC5
